# Photoaging: UV radiation-induced cGAS-STING signaling promotes the aging process in skin by remodeling the immune network

**DOI:** 10.1007/s10522-025-10268-1

**Published:** 2025-06-20

**Authors:** Antero Salminen, Kai Kaarniranta, Anu Kauppinen

**Affiliations:** 1 Department of Neurology, Institute of Clinical Medicine, University of Eastern Finland, P.O. Box 1627, 70211 Kuopio, Finland; 2 Department of Ophthalmology, Institute of Clinical Medicine, University of Eastern Finland, P.O. Box 1627, 70211 Kuopio, Finland; 3 Department of Ophthalmology, Kuopio University Hospital, P.O. Box 100, FI-70029 KYS Kuopio, Finland; 4 School of Pharmacy, Faculty of Health Sciences, University of Eastern Finland, P.O. Box 1627, 70211 Kuopio, Finland

**Keywords:** Ageing, Immunosuppression, Immunosenescence, Inflammaging, Inhibitory checkpoints

## Abstract

Excessive exposure of the skin to UV radiaton (UVR) accelerates the aging process and leads to a photoaging state which involves similar pathological alterations to those occurring in chronological aging. UVR exposure, containing both UVA and UVB radiation, triggers cellular senescence and a chronic inflammatory state in skin. UVR promotes oxidative stress and a leakage of double-stranded DNA (dsDNA) from nuclei and mitochondria into the cytoplasm of keratinocytes and fibroblasts. It is recognized that cytosolic dsDNA is a specific danger signal which stimulates cytoplasmic DNA sensors. The activation of the signaling through the cyclic GMP-AMP synthase (cGAS)-stimulator of interferon genes (STING) is a major defence and survival mechanism combatting against tissue injuries. There is abundant evidence that UVR exposure of skin stimulates cGAS-STING signaling which promotes cellular senescence and remodels both the local and systemic immune network. cGAS-STING signaling activates the IRF3 and NF-κB signaling pathways which trigger both pro-inflammatory and immunosuppressive responses. Moreover, cGAS-STING signaling stimulates inflammatory responses by activating the NLRP3 inflammasomes. Senescent fibroblasts secrete not only cytokines but also chemokines and colony-stimulating factors which induce myeloid differentiation and recruitment of immune cells into inflamed skin. Photoaging is associated with an immunosuppressive state in skin which is attributed to an expansion of immunosuppressive cells, such as Tregs. UVR-induced cGAS-STING signaling also stimulates the expression of PD-L1, a ligand for inhibitory immune checkpoint receptor, which evokes an exhaustion of effector immune cells. There is clear evidence that cGAS-STING signaling can also accelerate chronological aging by remodeling the immune network.

## Introduction

Repeated exposure of the skin to excessive sunlight is known to accelerate the chronological aging process in the skin (Gilchrest 2013; Rittie and Fisher 2015). Sunlight contains both UVA and UVB radiation (UVR) which induces a premature aging process, commonly called photoaging. Photoaging as well as the chronological aging process are associated with a remodeling of the immune system, i.e., the development of acute and chronic inflammatory responses and the counteracting immunosuppression are characteristic pathological alterations encountered in both the accelerated and normal aging process (Schwarz et al. 2008; Bernard et al. 2019; Salminen et al. 2022). There is abundant evidence that cellular senescence has a crucial role in the control of immune responses in skin during the photoaging and chronological aging processes (Fitsiou et al. 2021; Ho and Dreesen 2021). For example, UVR exposure induces both direct and indirect changes in nuclear and mitochondrial DNA and thus it triggers inflammatory changes which disturb dermal homeostasis. It is known that oxidative stress can disturb the integrity of nuclear DNA and the function of mitochondria leading to a leakage of double-stranded DNA (dsDNA) into the cytoplasm (Schuch et al. 2017; Li et al. 2023a; Cadet et al. 2024). Especially, UVR exposure triggers a release of mitochondrial DNA (mtDNA) into the cytoplasm. Currently, it is known that cytosolic dsDNA is a danger signal which activates cytoplasmic DNA sensors. The cyclic GMP-AMP synthase (cGAS)-stimulator of interferon genes (STING), i.e., cGAS-STING signaling, is a major defence mechanism triggered by cytosolic dsDNA (Motwani et al. 2019; Hopfner and Hornung 2020). The activation of cGAS-STING signaling stimulates the immune network through the interferon regulatory factor 3 (IRF3) and nuclear factor-κB (NF-κB)-driven signaling pathways. An activation of cGAS-STING signaling can induce both pro-inflammatory and immunosuppressive responses, as has been observed in the photoaging state. We will briefly introduce the function of cGAS-STING signaling and then examine in detail how UVR-activated cGAS-STING signaling induces cellular senescence in the skin and remodels the function of the immune network thus promoting a premature aging process in the sun-exposed skin.

## Photoaging

The aging process of the skin involves both intrinsic and extrinsic components, i.e., intrinsic aging describes the processes involved in chronological aging, whereas the extrinsic process refers to external events, such as sunlight-induced UVR exposure as well as the actions of environmental toxins and infections (Fisher et al. 2002; Durai et al. 2012; Cavinato and Jansen-Dürr 2017). Sunlight contains a component of UVB (280–320 nm) radiation which penetrates only into the epidermis, whereas UVA (320–400 nm) radiation can penetrate deeper and gain access to the dermis (Holick et al. 2016). The repeated exposure to sunlight, i.e., UVA and UVB radiation, induces pathological changes in the skin which resemble those encountered in the chronological aging process, thus this state has been called photoaging (Kligman 1986; Rittie and Fisher 2015). This means that photoaging represents an accelerated premature aging process and there are many review articles which have described the differences between the photoaging and chronological aging processes in the skin (Fisher et al. 2002; Durai et al. 2012; Rittie and Fisher 2015). Photoaging has been characterized by increased (i) the appearance of wrinkles and spider veins, (ii) epidermal atrophy and loss of elasticity, (iii) irregular pigmentation, freckles, and increased tanning, and (iv) early indications of skin cancer. A degradation of collagen and elastin fibers in the dermis is a hallmark of the pathology of photoaging. These histological lesions in the extracellular matrix (ECM) have been attributed to the significant accumulation of senescent fibroblasts within the dermal layer of photoaged skin (Fitsiou et al. 2021). The UVB irradiation of human dermal fibroblasts stimulates the expression of the members of the matrix metalloproteinases (MMP), especially MMP-1 and MMP-3, provoking the breakdown of connective tissue componens and thus enhancing tumor invasion and metastasis (Brenneisen et al. 2002). Senescent fibroblasts not only disturb the structure of the ECM but senescent cells can also trigger a low-grade inflammation and subsequently lead to the development of a counteracting immunosuppressive state in the sun-exposed skin (Schwarz 2008; Salminen et al. 2022; Zhou et al. 2024).

In the skin, excessive UVA and UVB exposures evoke diverse cellular stresses, i.e., oxidative stress and stresses in endoplasmic reticulum and mitochondria (Birch-Machin et al. 2013; Brand et al. 2018; Tai et al. 2024). Several investigators have demonstrated that oxidative stress is a major hallmark of both UVA and UVB irradiation in the skin as well as in isolated fibroblasts and keratinocytes (Cavinato and Jansen-Dürr 2017; Brand et al. 2018; Negre-Salvayre and Salvayre 2022; Calvo et al. 2024). Reactive oxygen species (ROS) can provoke cellular stress via several different mechanisms, e.g., by inducing damage in both genomic and mitochondrial DNA as well as by triggering oxidative alterations in both structural lipids and proteins. However, cells in the skin contain different antioxidants which strive to control the level of oxidative stress and thus defend against UVR-induced ROS generation (Calvo et al. 2024). Moreover, it has been reported that UVR treatment can induce the expression of antioxidative and anti-inflammatory enzymes, such as heme oxygenases (Chen et al. 2019). There is also abundant clinical evidence that diverse antioxidants, either topical or endogenous, can alleviate the signs of skin pathology in photoaging (Rabe et al. 2006; Calvo et al. 2024). The UVB-induced oxidative stress also induces disturbances in mitochondrial functions and triggers a leakage of mitochondrial DNA (mtDNA) from mitochondria (Birch-Machin et al. 2013; Newman and Shadel 2023). Accordingly, UVB-induced oxidative stress can damage and cause the fragmentation of nuclear DNA and subsequently promote a release of dsDNA fragments into the cytoplasm (Schuch et al. 2017; Cadet et al. 2024). A loss of nuclear and mitochondrial DNA integrity is a crucial feature of cellular senescence not only in accelerated photoaging but also in the chronological aging process (Lopez-Otin et al. 2023).

## cGAS-STING signaling

The accumulation of dsDNA into the cytoplasm is an important endogenous danger signal which activates cytosolic dsDNA sensors. There are different types of dsDNA sensors which can switch on specific signaling pathways not only to defend the cell itself but also to alert the immune system that there are tissue injuries (Briard et al. 2020). The cGAS-STING pathway is present in different tissues and cell types and its signaling mechanisms have been studied especially in infections as well as in many inflammatory diseases and cancers (Motwani et al. 2019; Hopfner and Hornung 2020). While there are also certain other dsDNA sensors, these seem to possess more specific functions and their expression patterns are more limited, e.g., the absent in melanoma 2 (AIM2) and the Toll-like receptor 9 (TLR9) (Jin et al. 2012; Chan et al. 2015). cGAS-STING signaling has a crucial role in the defence against viral infections (Ma and Damania 2016), although in many chronic inflammatory diseases it aggravates the pathogenesis, such as in atherosclerosis (Sakai et al. 2023) and rheumatoid arthritis (Zhu and Zhou 2024). Recent investigations have revealed that since cGAS-STING signaling induces cellular senescence, it can also promote many facets of the aging process (Lan et al. 2019; Loo et al. 2020; Gulen et al. 2023).

The cGAS-STING signaling pathway is highly conserved in vertebrates and it contains different phases, (i) the cyclic GMP-AMP synthase (cGAS) is a sensor for cytosolic dsDNA, (ii) soluble cGAMP dinucleotides are second messengers, (iii) cGAMP-activated STING receptors form signaling platforms in the Golgi complex, and (iv) the STING platforms interact with many signaling molecules, such as TANK-binding kinase 1 (TBK1), and (v) finally these interacting molecules trigger many downstream signaling pathways, such as the interferon regulatory factor 3 (IRF3) and nuclear factor-κB (NF-κB)-driven pathways (Motwani et al. 2019; Hopfner and Hornung 2020). The binding of dsDNA to the cGAS protein activates this synthase enzyme which catalyzes the formation of the cGAMP dinucleotide from ATP and GTP nucleotides. The cGAS enzyme is the rate limiting enzyme for the cGAS-STING signaling pathway and thus it can control many crucial cellular functions, e.g., senescence, autophagy, and DNA repair as well as important responses of the innate and adaptive immunity (Motwani et al. 2019; Liu et al. 2022). Given that the leakage of dsDNA into the cytoplasm is a danger signal, there also exist enzymes which can degrade cytosolic dsDNA and thus suppress the activation of cGAS-STING signaling. For instance, impairing the activity of the three prime repair exonuclease 1 (TREX1) and deoxyribonuclease 2 (Dnase 2) robustly augmented the level of cytosolic dsDNA, stimulated cGAS-STING signaling, and subsequently increased cellular senescence in different experimental models (Takahashi et al. 2018; Du et al. 2023). Because TREX1 is a direct inhibitor of dsDNA-driven cGAS-STING signaling, TREX1 is a novel immunotherapeutic target in many diseases (Hemphill et al. 2021).

The cGAMP dinucleotide acts as a second messenger which targets the STING dimers in the membranes of ER. The binding of the cGAMP molecule to the STING dimer leads to their oligomerization and trafficking to the Golgi complex where they form STING signaling platforms (Kato et al. 2017; Hopfner and Hornung 2020). The cGAMP dinucleotides not only function as intracellular messengers but they can also be secreted out of mouse and human cells via the volume-regulated anion channels (VRAC/LRRC8) (Zhou et al. 2020). Subsequently, the cGAMP dinucleotides can be imported via the solute carrier SLC19A1 protein and the anion channel LRRC8 into neighbouring cells where they can stimulate cGAS-STING signaling (Luteijn et al. 2019; Zhou et al. 2020). The intercellular cyclic dinucleotide-STING signaling mechanism is evolutionarily conserved since for instance, bacteria utilize this signaling pathway (Morehouse et al. 2020). Interestingly, the ectonucleotide pyrophosphatase/phosphodiesterase 1 (ENPP1) enzyme can cleave the extracellular cGAMP messenger molecule and thus it is able to inhibit the function of this intercellular immunotransmitter (Carozza et al. 2022). However, intercellular cGAMP transmission is an important mechanism in spreading cell intrinsic immunity into neighbouring cells, especially into immune cells where cGAMP can control immune responses (Ablasser et al. 2013; Jütte et al. 2021).

**Fig. 1:**
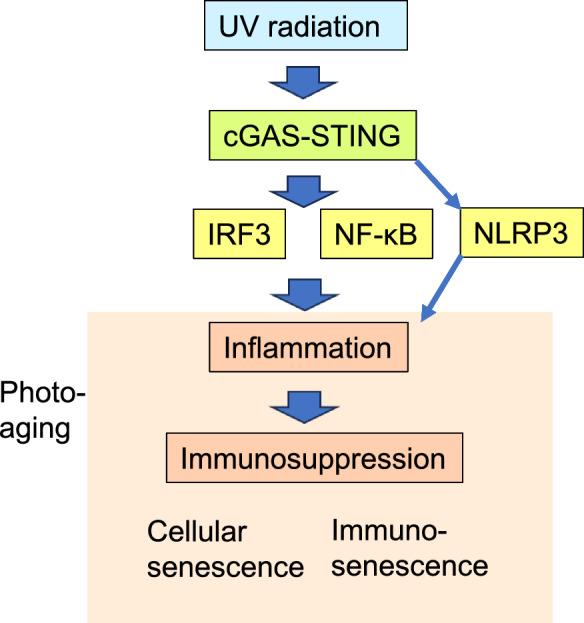
A schematic presentation of cGAS-STING-induced remodelling of the immune network in the sunlight-exposed skin. UVR stimulates cGAS-STING signaling which activates canonical signaling via the IRF3 and NF-κB pathways and non-canonical responses via the NLRP3 inflammasomes. Inflammation and immunosuppression are associated with cellular senescence and immunosenescence in photoaged skin. Abbreviations: *cGAS-STING*, cyclic GMP-AMP synthase-stimulator of interferon genes; *IRF3*, interferon regulatory factor 3; *NF-κB*, nuclear factor-κB; *NLRP3*, NLR family pyrin domain containing 3; *UV*, ultraviolet

The STING oligomeric platform interacts with many signaling molecules which trigger both canonical and non-canonical pathways (Balka and De Nardo 2021; Coderch et al. 2023). The IRF3 and NF-κB signaling pathways represent canonical STING pathways which stimulate not only immune responses but are also involved in many survival functions (Fig. 1). The IRF3 transcription factor is transported from the Golgi complex into the nuclei where it activates the expression of type-1 interferons (*IFN-α/β*) and many interferon-stimulated genes (ISG). Type-1 interferons, i.e., IFN-α and IFN-β, trigger the STAT1 and STAT2-driven transcription via the interferon receptors (IFNR1/2) inducing the expression of many immune and survival genes, especially antiviral genes (Platanias 2005; Rauch et al. 2013). NF-κB signaling is induced through the STING/IKKε/TAK1/IKKαβ pathway (Abe and Barber 2014; Balka and De Nardo 2021). In addition to being a major inducer of immune responses, the NF-κB system can also regulate other important cellular functions, such as apoptosis, cellular senescence, cell-cycle regulation, and cell survival (Yang et al. 2000; Salminen et al. 2012; Verzella et al. 2020). The STING signaling platform can also promote non-canonical functions; it can stimulate autophagy, activate NLRP3 inflammasomes, and trigger endoplasmic reticulum (ER) stress (Gaidt et al. 2017; Gui et al. 2019; Zhang et al. 2022; Liu et al. 2024). For instance, Zhang et al. (2022) demonstrated that in the ER, the activated STING protein interacted with the protein kinase R-like endoplasmic reticulum kinase (PERK) which subsequently phosphorylated the eukaryotic initiation factor 2α (eIF2α). STING-PERK-eIF2α signaling promoted cellular senescence and bleomycin-induced fibrosis in mouse lungs. Gui et al. (2019) reported that in many human cells, the STING platform also stimulated the non-canonical autophagy via the STING/WIPI2/ATG5 pathway. Interestingly, they also demonstrated that this non-canonical autophagy cleansed both the cytosolic dsDNA and the STING platforms, i.e., this represents a negative feedback response. It has been reported that activation of the cGAS-STING pathway can promote inflammatory responses by activating the NLRP3 inflammasomes (Gaidt et al. 2017; Wang et al. 2020; Xiao et al. 2023; Liu et al. 2024) (Fig. 1). For instance, the IRF3 transcription factor can transactivate the *NLRP3* gene (Xiao et al. 2023) and the STING protein can interact with the NLRP3 protein and thus activate the secretion of IL-1β (Wang et al. 2020). Moreover, Gaidt et al. (2017) demonstrated that cGAS-STING signaling stimulated a cell death program, i.e., the STING protein was transported into lysosomes where it induced a potassium efflux which subsequently activated the NLRP inflammasomes. In human myeloid cells, the activation of NLRP3 inflamasomes induced a pyroptotic cell death. These observations clearly indicate that the STING platform is a signaling hot spot in the Golgi complex.

## UVR stimulates cGAS-STING signaling in the skin

The cGAS-STING signaling pathway is not only an evolutionarily conserved defence mechanism against bacterial and viral infections but it also alerts from genotoxic and mitochondrial stresses which evoke a leakage of dsDNA into the cytoplasm (Hopfner and Hornung 2020; Newman and Shadel 2023; Patel et al. 2023). Diverse dysfunctions of mitochondria can induce a release of mitochondrial DNA (mtDNA) into the cytoplasm in immune and non-immune cells. Newman and Shadel (2023) have described several stresses which induce a leakage of mtDNA from mitochondria, e.g., ER stress, metabolic stresses, and an increase in mitochondrial generation of ROS, all of which are known to induce cellular senescence and promote the aging process. Moreover, a loss of integrity in genomic DNA can trigger a leakage of dsDNA into the cytoplasm. For instance, DNA replication stress and trafficking of transposons can increase an accumulation of cytosolic dsDNA (Saxena and Zou 2022; Mathavarajah and Dellaire 2024). In addition, disturbances in the integrity of the nuclear envelope enhance the release of genomic dsDNA fragments into the cytoplasm, e.g., in the Hutchinson-Gilford progeroid syndrome (Gonzalo et al. 2017), as well as in the chronological aging process (Sladitschek-Martens et al. 2022). On the other hand, cytoplasmic exonucleases, such as TREX1 and Dnase 2, can cleave cytosolic dsDNA and thus inhibit its ability to activate cGAS-STING signaling.

## UVR stimulates a leakage of dsDNA into the cytoplasm

There is convincing evidence that UVR, especially UVB exposure, induces both nuclear and mitochondrial DNA damage in the cells of the skin (Birch-Machin et al. 2013; Kciuk et al. 2020; Cadet et al. 2024). Sunlight, containing both UVA and UVB radiation, primarily induces indirect DNA lesions via the generation of ROS compounds which trigger oxidative stress (Schuch et al. 2017). UVR exposure elicits oxidative damage in DNA nucleotides (Cadet et al. 2024), e.g., the chronic exposure to UVB induced the generation of 8-oxo-7,8-dihydroguanine (8-oxoG) in mouse skin and subsequently enhanced carcinogenesis (Kunisada et al. 2005). An accumulation of 8-oxoG within tissues is a characteristic hallmark of the aging process and is also encountered in many age-related diseases (Radak and Boldogh 2010; Zhang and Li 2020). Moreover, in mouse fibroblasts and keratinocytes, UVR is able to stimulate a generation of different transposons, e.g., SINEs and LINE-1 (Wang et al. 1999; Touma et al. 2023). Furthermore, in human keratinocytes, UVB exposure also transcriptionally activated endogenous retroviral sequences (Hohenadl et al. 1999). There are many investigations which have indicated that UVR exposure also impairs the structure and permeability of the nuclear envelope (Zierler et al. 2006; Li et al. 2020). Li et al. (2020) demonstrated that when mouse skin was irradiated with UVB, this induced a rupture of the nuclear envelope in neutrophils. This effect was attributed to the disassembly of lamin B structure leading to the formation of neutrophil extracellular traps, i.e., NETosis (Vorobjeva and Chernyak 2020). There is clear evidence that UVR-induced damage and fragmentation of genomic DNA as well as a rupture of the nuclear envelope can lead to apoptosis, e.g., in human keratinocytes and T-lymphocytes (Bazar and Deeg 1992; Aufiero et al. 2006). Li et al. (2021) demonstrated that the UVB-induced DNA damage in human keratinocytes triggered apoptosis of these cells via the activation of the cGAS-STING signaling pathway.

When exposured to UVR, the mitochondria of the dermal cells undergo a functional decline and suffer structural damages (Juge et al. 2016; He et al. 2022; Li et al. 2023a, 2023b). Juge et al. (2016) revealed that UVB irradiation triggered mitochondrial fragmentation in human epidermal keratinocytes. Li et al. (2023a) reported that UVB exposure also increased the fission of mitochondria and induced a leakage of mtDNA into the cytoplasm in human keratinocytes (HaCaT). They demonstrated that the UVB-induced impairment of mitophagy caused a release of mtDNA into the cytosol in HaCaT cells. A decline in mitophagy after UVB irradiation resulted in a down-regulation of PINK1/parkin proteins, the major regulators of the mitophagy pathway. Moreover, Cavinato et al. (2024) demonstrated that the BNIP3L/NIX-mediated mitophagy pathway eliminated damaged mitochondria in UVB-treated human dermal fibroblasts. Interestingly, an increase in the level of cytosolic mtDNA in human keratinocytes stimulated STING signaling and subsequently activated a pro-inflammatory response mediated via the NLRP3 inflammasomes (Li et al. 2023a, 2023b). These studies clearly indicate that UVB exposure robustly inhibits the elimination of dysfunctional mitochondria leading to a leakage of mtDNA and an activation of cGAS-STING signaling.

## UVR stimulates cGAS-STING signaling in the skin

There are several reports demonstrating that the UVB-induced leakage of dsDNA into the cytoplasm can stimulate the cGAS-STING pathway in human and mouse keratinocytes and skin (Kemp et al. 2015; Sontheimer et al. 2017; Skopelja-Gardner et al. 2020; Li et al. 2021, 2023a, 2023b; Zuo et al. 2024) (Fig. 1). These findings have been confirmed by either pharmacological or knockout techniques, i.e., UVB exposure stimulated the activation of the cGAS receptor and subsequently triggered STING signaling (Li et al. 2023a; Sontheimer et al. 2017; Zuo et al. 2024). For instance, Zuo et al. (2024) demonstrated that the UVB-induced symptoms of photoaging were clearly attenuated in the skin of STING knockout mice. They also reported that salvianolic acid A, an anti-inflammatory stilbenoid compound, inhibited the activation of cGAS-STING signaling in skin macrophages and thus alleviated the severity of photodamage and tissue injuries. This highlights that cGAS-STING signaling in skin macrophages can induce photodamage and enhance the photoaging process in that tissue. Interestingly, Li et al. (2024) demonstrated that UVB-irradiated human keratinocytes secreted extracellular vesicles (EV) which triggered a STING- and inflammasome-mediated proinflammatory response in cultured human macrophages. It is known that UVB-treated keratinocytes secrete EVs containing many inflammatory factors which exert a bystander response to remodel the immune microenvironment in the photoaging process (Frommeyer et al. 2022). This indicates that UVB-induced photodamage in the epidermis can be transferred into dermal cells. Currently, the content of the EVs which can trigger the STING-mediated activation of macrophages needs to be clarified. Jütte et al. (2021) demonstrated that cGAMP dinucleotides generated by UVR-induced activation of cGAS enzyme were transported, as either soluble or EV-bound compounds, between cells in the mouse skin, inducing UVR-dependent autoimmune dermatitis. In tumor biology, it is known that cGAMP as well as DNA fragments can be transported in EVs from cancer cells into myeloid cells where they activate the STING pathway (Mekers et al. 2022). These observations indicate that cGAS-STING signaling can propagate photodamage from epidermal keratinocytes into healthy dermal cells promoting the photoaging process in the skin.

It is known that cGAS-STING signaling can induce both canonical and non-canonical immune responses, as described above. It now seems clear that UVR-induced cGAS-STING signaling in the skin can stimulate both the IRF3-mediated type-1 IFN response and the diverse immune responses driven by NF-κB (Kemp et al. 2015; Sontheimer et al. 2017; Skopelja-Gardner et al. 2020; Li et al. 2021) (Fig. 1). An activation of STING signaling was observed to stimulate the expression of IFN-α and IFN-β via the TBK1-IRF3 pathway in human keratinocytes and THP-1 monocytes (Kemp et al. 2015; Li et al. 2021). Skopelja-Gardner et al. (2020) examined cGAS knockout mice and they demonstrated that the UVB-induced IFN response was significantly dependent on cGAS signaling in mouse skin, whereas the UVB-related upregulation of TNF-α and IL-6 cytokines was independent of the cGAS receptor. Li et al. (2023b) reported that UVB exposure of human keratinocytes stimulated the expression of IL-1β via the activation of NLRP3 signaling, a non-canonical target of STING signaling. Moreover, Skopelja-Gardner et al. (2020) demonstrated that UVB irradiation of mouse skin triggered an extracellular release of cGAMP which augmented the level of the type-1 IFN response in mouse skin. They also revealed that the cGAS-mediated type-1 IFN response not only appeared in the expression of many ISG proteins in the skin but it could also be detected in the peripheral blood cells and in the kidney. Type-1 IFNs have an important regulatory role in the pathogenesis of many inflammatory diseases (Ji et al. 2023). Similarly, type-1 IFNs have crucial functions in the pathogenesis of several skin diseases, such as psoriasis (Gu et al. 2022; Xu et al. 2024), cutaneous lupus erythematosus (Wenzel et al. 2005), and dermatomyositis (Hile and Werth 2025).

## UVR-induced cGAS-STING signaling stimulates cellular senescence in the skin

There is convincing evidence that an exposure of the skin to both UVR and ionizing radiation can induce nuclear and mitochondrial damage and thus trigger a leakage of dsDNA, which as stated above, then stimulates cGAS-STING signaling and acts as a trigger for cellular senescence (Yang et al. 2017; Freyter et al. 2022; Touma et al. 2023; Cavinato et al. 2024; Zuo et al. 2024). It has been demonstrated that a more severe irradiation induces cGAS-STING-mediated apoptotic cell death in the skin (Kemp et al. 2015; Li et al. 2021). It is known that cGAS-STING signaling not only stimulates immune responses but it can also regulate the pathological state of cells, i.e., either inducing cellular senescence or causing cell death processes via apoptosis, ferroptosis, necroptosis, and pyroptosis (Murthy et al. 2020; Zhang et al. 2021; Liu et al. 2024). For instance, an inhibition of the activities of certain cytoplasmic DNAases, e.g., TREX and Dnase 2, robustly increased the amount of cytosolic dsDNA and consistently induced cellular senescence (Du et al. 2023; Techer et al. 2024). Accordingly, an increase in the level of cytosolic dsDNA either by inhibiting mitophagy or inducing replicative arrest, activated cGAS-STING signaling and then stimulated cellular senescence in different cell types (Lan et al. 2019; Zhong et al. 2022). Nonetheless, the mechanisms underpinning cGAS-STING-induced cellular senescence still need to be clarified. For instance, the IRF3 transcription factor can promote cellular senescence by transactivating the *p53* and *RB* genes, major players in cellular senescence (Moiseeva et al. 2006; Wu et al. 2024). IRF3 signaling also stimulated the expression of IFNα/β which can remodel immune responses and stimulate cellular senescence by enhancing the pro-inflammatory senescence-associated secretory phenotype (SASP) (Wang et al. 2024). The NF-κB signaling pathway, another downstream pathway of cGAS-STING signaling, is a major inducer of inflammatory cytokines secreted by senescent cells (Salminen et al. 2012). There is robust evidence that an activation of STING signaling promotes cellular senescence and induces age-related tissue degeneration through NF-κB signaling, e.g., in progeroid models (Tilstra et al. 2012). Given that STING signaling can activate ER stress via the non-canonical PERK pathway, it is not surprising that it is also a potent inducer of ER stress-stimulated cellular senescence in many pathological processes (Zhang et al. 2022; Dong et al. 2024).

An accumulation of senescent cells is a hallmark of the chronological aging process not only in the skin but also in many other tissues (Ho and Dreesen 2021; Lee et al. 2021; Franco et al. 2022; Lopez-Otin et al. 2023; Yu et al. 2025). Fitsiou et al. (2021) claimed that cellular senescence and its proinflammatory properties, i.e., the SASP, were the drivers of skin photoaging. UVR exposure stimulated a premature accumulation of senescent cells within the epidermis and dermis of the photoaged skin (Fitsiou et al. 2021; Yu et al. 2025). However, there are recent evidence indicating that after UVB exposure there were clear differences in the appearance of senescent cells between different cell types and between the epidermis and dermis (Yu et al. 2025). In the epidermis, melanocytes were more prone than keratinocytes to exhibit a senescence phenotype (Victorelli et al. 2019; Fitsiou et al. 2021) although after UVA and UVB exposure, cultured human keratinocytes displayed common markers of cellular senescence (Valerio et al. 2021; Bauwens et al. 2023). Human dermal fibroblasts are a cell type extensively used as a cellular senescence model in vitro as well as in UVA and UVB experiments. Yu et al. (2025) used single-cell RNA-sequencing from isolated skin samples to investigate the effects of long-term exposure to sunlight on cellular senescence in the epidermal and dermal cells in aged humans. They reported that sunlight-exposed senescent melanocytes displayed a robust elevation of melanin synthesis and enhanced pigmentation, whereas the senescent fibroblasts in the dermis exhibited a clear decrease in the synthesis of collagen and elastin fibers. They also reported that the expression of pro-inflammatory factors was significanly increased in senescent fibroblasts. Thus, it seems that fibroblasts have a crucial role in the photoaging process affecting not only the structure of ECM but also disturbing the immune properties of the photoaged skin.

In addition to being versatile regulators of the ECM structures, tissue fibroblasts possess many immune properties controlling both pro-inflammatory and immunosuppressive functions (Salminen et al. 2024). There are many single-cell investigations aiming to elucidate the properties of senescent fibroblasts in the skin of aged humans (Sole-Boldo et al. 2020; Zou et al. 2021; Zorina et al. 2022). All these human studies as well as those conducted in mice (Salzer et al. 2018) have detected a robust increase in the pro-inflammatory properties of dermal fibroblasts, such as elevated secretion of many interleukins, chemokines, and proteolytic enzymes. Zou et al. (2021) demonstrated that with aging in human skin there was a progressive accumulation of senescent cells and the appearance of chronic low-grade inflammatory state, i.e., the same alterations occurring in the photoaged skin. Their results also emphasized the importance of dermal fibroblasts in the aging process of the skin. Sole-Boldo et al. (2020) revealed that with aging human dermal fibroblasts, even those located in sunlight-protected area, gradually lost the properties of their typical cellular identity, i.e., many pro-inflammatory SASP factors were upregulated whereas cell–cell interactions had declined. It seems that an age-related senescence of fibroblasts not only disturbs the maintenance of ECM but it also remodels the immune microenvironment in the aged skin.

## cGAS-STING signaling remodels the immune network in the skin

A leakage of dsDNA into the cytoplasm is an alarming factor which activates DNA sensors to mount an immune defence against the perpetrator. The cGAS-STING signaling pathway alerts the immune system which generates pro-inflammatory responses in the acute phase, followed by an immunosuppressive state (Fig. 1). Interestingly, it is known that both the inflammatory phase and subsequently this more permanent immunosuppressive state are associated with the pathogenesis of photoaging (Schwarz 2008; Salminen et al. 2022). cGAS-STING signaling is crucial in this phenomenon since in this way UVR triggers the presence of cellular senescence which is able to remodel the immune system in many pathological states. For instance, it induces a pro-inflammatory SASP state which not only triggers inflammation in the affected tissue but by secreting cytokines, chemokines, and colony-stimulating factors, it can stimulate the immune network and recruit immune cells, e.g., immunosuppressive cells, into the inflamed tissue. This means that senescent cells have a key role in remodeling the immune network in photoaging and also during the chronological aging process (Salminen 2025).

## Pro-inflammatory signaling

The photoaging process involves three different immune states, i.e., (i) the early vasodilatory phase during which there is mast cell degranulation, dermal edema, erythema, and an increased sensitivity to pain, (ii) the inflammatory phase involving the infiltration of neutrophils, monocytes, and T lymphocytes as well as the secretion of proinflammatory mediators, and finally (iii) the resolution and immunosuppressive phase associated with many counteracting immunosuppressive responses and tissue repair processes (Terui et al. 2001; Motwani et al. 2017). Cellular senescence is a hallmark of the UVR-induced photoaging state and a driver of the pathogenesis of photoaging, in a similar manner as occurs in the chronological aging of the skin as well as in many age-related skin pathologies (Chin et al. 2023). It has been known for decades that UVR-induced DNA damage triggers a cellular stress which evokes inflammatory responses in the skin via the activation of NF-κB signaling and the NLRP3 inflammasomes (Feldmeyer et al. 2007; Tanaka et al. 2010; Hasegawa et al. 2016; Salminen et al. 2022) (Fig. 1). For example, Hasegawa et al. (2016) reported that UVR treatment of human keratinocytes led to an activation of NLRP3 inflammasomes promoting the secretion of IL-1β as well as increasing the secretion of many other inflammatory mediators, such as IL-1α, IL-6, TNF-α, and PGE2. They also revealed that UV-irradiation of human skin induced the expression of the NLRP3 protein in the epidermal layer. Moreover, Dawes et al. (2014) applied a genome-wide transcriptional profiling technique and demonstrated that UVB exposure of human skin induced a clear increase in the expression of many chemokines, e.g., CCL3, CCL4, CCL20, CXCL1, CXCL3, and CXCL5. Surowiak et al. (2014) reported that human keratinocytes and dermal fibroblasts isolated from the sunlight-exposed skin displayed a robust increase in the expression of COX-2 as compared to cells from sunlight-unexposed skin indicating that prostanoids were involved in the photoaging process. It is known that the expression of COX-2 is significantly upregulated in senescent cells and for instance, PGE2 can regulate the composition of the SASP secretome (Goncalves et al. 2021). All these results indicate that it is the pro-inflammatory phenotype of senescent cells which has a crucial role in the pathogenesis of photoaging in the skin.

There is abundant evidence that the cGAS-STING pathway is a potent inducer of pro-inflammatory responses via the STING-mediated canonical IRF3 and NF-κB signaling pathways as well as via the non-canonical activation of the NLRP3 inflammasomes (Fig. 1). For instance, it was observed that the activation of cGAS-STING signaling in senescent cells stimulated the NF-κB system which as described above, has a crucial role in the secretion of pro-inflammatory SASP factors, such as cytokines, chemokines, and matrix metalloproteinases (Chien et al. 2011; Salminen et al. 2012; Guo et al. 2021; Herbstein et al. 2024). In cooperation with other signaling pathways, the NF-κB system can regulate metabolic pathways and many functions of both innate and adaptive immunity (Oeckinghaus et al. 2011; Capece et al. 2022). Consequently, the cytokines and chemokines secreted from senescent cells are able to remodel the inflammatory microenvironment by regulating the functions of immune cells via their specific receptors. Moreover, NF-κB signaling can promote inflammatory responses by priming the NLRP3 inflammasomes, i.e., it led to the expression of the NLRP3 protein and the proforms of the IL-1β and IL-18 cytokines (Bauernfeind et al. 2009). The STING protein also stimulates the NLRP3 inflammasomes via certain non-canonical mechanisms, as described above. The IRF3/type-1 IFN pathway is another canonical cGAS-STING signaling route which also acts as a trigger of pro-inflammatory responses (Fig. 1). IFN-α and IFN-β have an important role in diverse inflammatory states, e.g., secreted IFN cytokines activate tissue macrophages thus augmenting the inflammatory state in autoimmune diseases, whereas an excessive IFN signaling can lead to interferonopathies (Siebeler et al. 2023; Mendonca and Fremond 2024). Type-1 IFNs have also many specific effects on the function of different immune cells, such as macrophages, neutrophils, natural killer cells, and T lymphocytes, and in that way they control the pathogenesis of many immune diseases (Zannikou, et al. 2024). Hile et al. (2020) have examined the many ways in which the type-1 IFNs exert influences on healthy and diseased skin. It seems that many cellular stresses not only viral and bacterial infections stimulate cGAS-STING signaling which activates immune responses to restore homeostasis within the inflamed tissue.

## Immunosuppressive responses

Over 40 years ago, it was observed that the UVR-induced inflammatory phase was associated with a chronic immunosuppressive state which involved both a local immunosuppression and also a systemic immunosuppressive response (Noonan and De Fabo 1990; Schwarz 2008; Bernard et al. 2019) (Fig. 1). It has now become evident that the immunosuppressive state is mainly induced by a robust expansion and an increased activity of immunosuppressive regulatory T (Treg) cells (Schwarz 2005; Maeda et al. 2008). In the skin, Tregs are tissue-resident immune cells which have a key role in the maintenance of tissue homeostasis, e.g., in cutaneous wound healing (Nosbaum et al. 2016; Ali and Rosenblum 2017). There is a close cooperation between tissue specific Langerhans cells (LC) and Tregs in the generation of the immunosuppressive state. After UVR exposure, LCs emigrate into the regional lymph nodes where they present antigens to Treg cells which subsequently traffic into the affected skin (Schwarz et al. 2010; Price et al. 2015). Tregs have many important functions in UVB-treated skin, (i) they enhance the resolution of inflammation, (ii) they improve the repair process of tissue injuries, and finally (iii) they suppress the contact hypersensitivity in the skin. Although Tregs are the main source of immunosuppression in the photoaged skin, there are investigations indicating that UVR exposure also increases the recruitment of myeloid-derived suppressor cells (MDSC) into inflamed skin (Liang et al. 2017). Moreover, in the photoaged skin, the presence of immunosuppressive cells also augments the immunosuppressive properties of other immune cells, such as natural killer T cells (NKT), M2 macrophages, and regulatory B cells (Breg) (McKee et al. 2014; Liu et al. 2018; Li et al. 2022). It is known that immunosuppressive cells inhibit the function of many immune effector cells, such as T, B, and NK cells, thus leading to their immunosenescence (Ye et al. 2012; Salminen 2021a). It should be remembered that immunosuppressive cells secrete many factors, e.g., IL-10, TGF-β, and ROS compounds, which disturb tissue homeostasis and promote cellular senescence and the aging process (Tominaga and Suzuki 2019; Salminen 2021b).

There has been considerable interest in elucidating the mechanisms which induce the expansion of skin immunosuppressive Tregs during the photoaging process. Given that there is abundant evidence that UVB-induced photoaging is associated with an accumulation of cytosolic dsDNA and the activation of cGAS-STING signaling, it seems probable that STING signaling is involved in the differentiation and activation of Treg cells. Interestingly, there is novel evidence indicating that type-1 interferons can induce the differentiation and survival of the immunosuppressive Treg cells (Field et al. 2020; Vitale et al. 2021; Fueyo-Gonzalez et al. 2022; Nishiyama et al. 2022; Eskandari et al. 2023). For example, Vitale et al. (2021) reported that type-1 IFNs induced the differentiation of peripheral Treg cells and IFN-α inhibited the expansion of pro-inflammatory Th17 cells. These alterations enhance immunosuppression in the balance between Th17/Treg cells in mice. Fueyo-Gonzalez et al. (2022) demonstrated that IFN-β stimulated the induction of mouse Treg cells by activating STAT1 signaling which induced the expression and acetylation of the FOXP3 transcription factor, a master regulator of the immunosuppressive activity of Tregs. Accordingly, Metidji et al. (2015) reported that the knockout of the type-1 IFN receptor, i.e., the IFNAR1 protein, suppressed the expansion of Treg cells and development of an immunosuppressive state in mouse inflammatory conditions. There are also observations that the activation of the intrinsic STING signaling in T cells can stimulate the expression of the human FOXP3 protein and subsequently induce immunosuppression in an IFN-independent manner (Ni et al. 2022; Lin et al. 2024). Lin et al. (2024) demonstrated that an activation of STING signaling in T cells triggered the STING-MAPK-CREB transactivation pathway which triggered the differentiation of Treg cells in mice. There are many indications that it is not only the differentiation of Tregs but STING signaling can also promote tissue immunosuppression via the expansion of M2 macrophages and MDSCs (Günthner and Anders 2013; Liang et al. 2017; Chistiakov et al. 2018). For instance, the IRF3 transcription factor can induce the differentiation of monocytes into macrophages and subsequently polarize them into the M2 immunosuppressive phenotype (Chistiakov et al. 2018). Moreover, Liang et al. (2017) revealed that the X-ray irradiation of mice induced tissue immunosuppression through the recruitment of MDSCs into the radiation site via the CCR2-STING signaling pathway. These studies indicate that by activating a network of immunosuppressive cells, cGAS-STING signaling is a potent inducer of tissue immunosuppression in the presence of different insults.

It seems that UVB-induced cellular senescence in the skin has an important role not only in eliciting pro-inflammatory responses but also in the generation of the immunosuppressive state. The activation of cGAS-STING signaling stimulates the production and secretion of type-1 IFNs and many chemokines which subsequently trigger the recruitment and differentiation of immunosuppressive cells to counteract inflammation with the ultimate goal of achieving the resolution of inflammatory state. In addition, senescent cells can promote the immunosuppressive state in inflamed skin by expressing the ligands for the inhibitory immune checkpoint receptors present in many immune cells, such as in the surveying cytotoxic CD8^+^ T cells, NK cells, and macrophages (Onorati et al. 2022; Wang et al. 2022; Majewska et al. 2024; Salminen 2024a). The programmed cell death protein-1 (PD-1)/ligand-1 (PD-L1) checkpoint pathway is a well-recognized inhibitory checkpoint signaling pathway (Ghosh et al. 2021; Beenen et al. 2022). However, it is known that senescent cells also express several other ligands for the diverse inhibitory checkpoint receptors present in immune cells (Salminen 2024b). The ligands for inhibitory checkpoint receptors are “don’t eat me” markers and thus senescent cells not only prevent their immune elimination but they can also inhibit the function of immune cells via these inhibitory receptors (Buckle and Guillerey 2021; Baldanzi 2022; Salminen 2025). The PD-L1 protein is expressed in all major cells in the skin, i.e., keratinocytes, Langerhans cells, melanocytes, and dermal fibroblasts (Tanaka et al. 2022; Majewska et al. 2024; Zhou et al. 2024). There is clear evidence that UVB exposure robustly increases the expression of the PD-L1 protein in human and mouse skin (Zhu et al. 2018; Wang et al. 2019; Dickinson et al. 2021; Zhou et al. 2024). Given that cGAS-STING signaling is a potent inducer of the expression of the PD-L1 protein (Cheng et al. 2020; Du et al. 2022), it seems probable that UVB treatment promotes immunosuppression in the skin via the inhibitory immune checkpoints. Zhou et al. (2024) demonstrated that the human sunlight-exposed skin compared to unexposed skin displayed a significant enrichment of PD-L1 expression. The enhanced expression of PD-L1 in photoaged skin indicates that inhibitory PD-1/PD-L1 checkpoint signaling has an important role in the generation of the immunosuppressive state within the skin. Moreover, there is a close cooperation between the PD-1/PD-L1 signaling and the immunosuppressive network promoting the immunosuppressive state in the aged skin (Salminen 2025). It appears that a senescence state of the skin induced by UVR exposure remodels the immune network leading to the development of the immunosuppressive microenvironment in photoaged skin.

## Does cGAS-STING signaling promote the chronological aging process?

The primary cause of photoaging involves UVR-induced genotoxic changes in nuclear integrity and a disruption of mitochondrial homeostasis. Similar disturbances have been observed during the chronological aging process (Lopez-Otin et al. 2023). There is abundant evidence that the increased level of cytosolic dsDNA, e.g., after a leakage of mtDNA, promotes inflammatory responses within aged tissues (Zhong et al. 2022; Newman and Shadel 2023; Ding et al. 2024). Age-related leaky mitochondria have been associated with a decline in mitophagy in both immune and non-immune cells (Zhong et al. 2022; Ding et al. 2024; Jimenez-Loygorri et al. 2024). The theory that DNA damage lay at the core of the aging process was presented over fifty years ago (Alexander 1967) and later this hypothesis has received mounting support indicating that indeed, the integrity of genomic DNA is lost with aging (Schumacher et al. 2021). As a proof of principle about the alarming role of dsDNA, it has been demonstrated that the cytoplasm contains many exonucleases, e.g., TREX1 and DNase2 (Takahashi et al. 2018), as well as a number of dsDNA sensors, such as cGAS-STING, which activate both the innate and adaptive immune systems. Currently, there is clear evidence that the cGAS-STING pathway not only promotes the UVR-induced photoaging but it also has an important role in the chronological aging process, especially in age-related diseases (Paul et al. 2021; Gulen et al. 2023; Schmitz et al. 2023; Zheng et al. 2023).

It does seem that an accumulation of senescent cells within tissues is a common fundamental property underpinning both the photoaging and chronological aging process. Senescent cells exhibit a pro-inflammatory secretory phenotype, i.e., SASP, which promotes both acute inflammatory responses as well as a chronic counteracting immunosuppressive state. It is known that cGAS-STING signaling stimulates cellular senescence in both photoaging and chronological aging (Yang et al. 2017; Schmitz et al. 2023; Herbstein et al. 2024; Zuo et al. 2024). Senescent cells trigger pro-inflammatory responses leading to a state called inflammaging in the chronological aging process (Franceschi et al. 2000). Moreover, the NF-κB and type-1 IFN signaling pathways, probably induced by the activation of STING, have a key role in the generation of the SASP state in both photoaging and chronological aging (Benayoun et al. 2019; Cao 2022; Capece et al. 2022; Rasa et al. 2022). The SASP factors secreted by senescent cells include diverse cytokines, chemokines, growth factors, and metalloproteinases, which means that these cells can (i) remodel the immune network (Freund et al. 2010; Wang et al. 2022), (ii) reshape the ECM (Mavrogonatou et al. 2023) and (iii) expand senescence into neighbouring cells via the secretion of extracellular vesicles (Takasugi 2018). Interestingly, senescent cells not only induce pro-inflammatory responses but they can also trigger the counteracting immunosuppressive state by secreting colony-stimulating factors and chemokines which enhance the differentiation and/or recruitment of immunosuppressive cells into inflamed tissues. As described above, the similar processes occur in both photoaging and chronological aging. For example, a significant expansion of Tregs and MDSCs has been observed in both photoaging and in the chronological aging process (Maeda et al. 2008; Ruhland et al. 2016; Liang et al. 2017; Salminen 2020). Moreover, the immunosuppressive state is accentuated in many age-related diseases (Deng et al. 2022). Senescent cells also expand tissue immunosuppression through the expression of “don’t eat me” ligands, such as the PD-L1 proteins, which are known to enhance immunosenescence in both photoaging and chronological aging (Wang et al. 2019; Dickinson et al. 2021; Onorati et al. 2022; Salminen 2025). All these investigations clearly indicate that photoaging and chronological aging not only promote cellular senescence but they can also remodel the immune network. It is likely that in both accelerated and lifelong aging processes, the cGAS-STING-mediated remodeling of the immune network is a driving mechanism leading to immunosenescence and the degeneration of tissues (Fig. 1).

## Conclusions

There is abundant evidence that UVR stimulates oxidative stress in the skin although the pathogenesis of photoaging still needs to be clarified. Recent investigations have revealed that cytosolic dsDNA has a crucial role not only in the chronological aging process but especially in the pathogenesis of photoaging. For many decades, it has been known that the UVR exposure of the skin evokes DNA damage in both nuclei and mitochondria and in addition, it disturbs the integrity of the nuclear envelope and mitochondrial membranes thus promoting a leakage of dsDNA into the cytoplasm. Currently, it is recognized that cytosolic dsDNA is a powerful danger signal activating cytoplasmic DNA sensors. The cGAS-STING signaling is a potent alerting mechanism which by activating the IRF3 and NF-κB signaling pathways, not only enhances cell survival but also stimulates inflammatory responses within the sunligh-exposed skin. Photoaging is also associated with an accumulation of senescent dermal fibroblasts which subsequently enhance the remodeling of the immune network in the skin. There is clear evidence that photoaging is associated with an immunosuppressive state in the skin. This has been attributed to an expansion of immunosuppressive cells in the skin, especially Tregs and M2 macrophages. Interestingly, the activation of cGAS-STING signaling also stimulates the expression of the ligands for many inhibitory checkpoint receptors, such as PD-1/PD-L1 signaling. These ligands prevent the immune elimination of senescent fibroblasts and thus promote the aging process. The activation of inhibitory checkpoint receptors also exhaust effector immune cells thus enhancing immunosenescence in the skin. There are several indications that the effects of photoaging are not restricted into the sunlight-exposed skin but it has impacts on the whole-body aging process (Franco et al. 2022). Currently, there are different drug discovery approaches targetting the cGAS-STING pathway, e.g., several inhibitors of the cGAS enzyme and the activity of the STING platform (Decout et al. 2021).

## Declarations

### Data availability

No datasets were generated or analysed during the current study.
